# α-Crystallin distribution in retinal pigment epithelium and effect of gene knockouts on sensitivity to oxidative stress

**Published:** 2007-04-04

**Authors:** Jennifer Yaung, Manlin Jin, Ernesto Barron, Christine Spee, Eric F. Wawrousek, Ram Kannan, David R. Hinton

**Affiliations:** 1 Department of Pathology, Keck School of Medicine of the University of Southern California, Los Angeles, CA; 2 Ophthalmology, Keck School of Medicine of the University of Southern California, Los Angeles, CA; 3 Arnold and Mabel Beckman Macular Research Center, Los Angeles, CA; 4 Doheny Eye Institute, Los Angeles, CA; 5 National Eye Institute, DHHS, Bethesda, MD

## Abstract

**Purpose:**

To investigate the susceptibility of retinal pigment epithelium (RPE) from αA (-/-) and αB (-/-) mice to oxidative stress, and the subcellular changes of αA and αB-crystallins under oxidative stress.

**Methods:**

The effect of hydrogen peroxide (H_2_O_2_) on apoptosis in RPE from αA (-/-), αB (-/-), and wild type (wt) mice was assessed by TUNEL and AnnexinV/Propidium Iodide assays. H_2_O_2_-induced changes in caspase-3 activity and mitochondrial permeability transition (MPT) were determined. Human RPE in early passages (2-4) were starved in 1% FBS-containing Dulbecco's modified Eagle medium (DMEM) and treated with H_2_O_2_ for 24 h. Gene expression was quantitated by real time PCR. Confocal microscopy was used to examine α-crystallin compartmentalization. Whole cell and mitochondrial α-crystallin protein amounts were examined by transmission electron microscopy (TEM) and Western blot analysis.

**Results:**

RPE from αA (-/-), αB (-/-) mice exhibited increased susceptibility to apoptosis induced by H_2_O_2_, increased caspase-3 activation, and increased MPT. Treatment of human RPE with H_2_O_2_ resulted in a dose-dependent decrease in αB-crystallin mRNA expression. Confocal microscopy and subcellular fractionation of RPE showed that H_2_O_2_ treatment decreased cytosolic and mitochondrial pools of αB-crystallin but caused no change in αA-crystallin content. TEM confirmed changes in expression of αA and αB-crystallins with oxidative stress.

**Conclusions:**

Lack of α-crystallins renders RPE cells more susceptible to apoptosis from oxidative stress. Mitochondrial α-crystallins may play an important role in the protection from increased susceptibility of RPE in oxidative stress.

## Introduction

α-Crystallins belong to the family of small heat shock proteins (sHSPs) that include Hsp25 and Hsp27 [1]. While original studies on α-crystallins dealt with their abundant expression and role in the lens, it is now generally accepted that α-crystallins are proteins with entirely different non-lens roles [2,3] and expressed in multiple tissues of the body [4]. The two forms of α-crystallins, A and B, share an amino acid sequence homology of about 57% and are found in heterogeneous aggregates of the two proteins [5]. While the distribution of αA and αB-crystallins are different-αA is found predominantly in the lens while αB is ubiquitous-both have been demonstrated to protect cells from thermal and metabolic stress [6]. Furthermore, their ability to prevent apoptosis by inhibiting caspases implies that αA and αB-crystallins may provide crucial physiological functions in non-lens tissues [7].

An analysis of the expression of crystallins in the mouse retina showed that αA, αB, β, and γ-crystallins were found in the inner and outer nuclear layers and the retinal pigment epithelium (RPE) [8]. In another study, RPE cells overexpressing αB-crystallin showed resistance to apoptosis, suggesting that α-crystallins may play a beneficial role in preventing stress-induced cell death [9]. While both αA and αB-crystallins offer cell protection, the relative potencies and mechanisms of cell protection have not been fully delineated. Andley et al. [10] found that the antiapoptotic activity of αA is greater than αB-crystallin in the lens, while Mao et al. [11] have shown that αA and αB-crystallins display similar degrees of protection against apoptosis in both lens and non-lens tissues. Information on the subcellular localization of αA and αB-crystallin within RPE, which has hitherto not been studied in detail, may offer clues for similarities/dissimilarities in α-crystallin function.

Generation of mice lacking αA (αA(-/-)) and αB (αB(-/-))-crystallin has provided valuable insights into the functional roles of these proteins in the lens. Lenses of αA-crystallin deficient mice appeared structurally normal, but developed opacification quickly with age [12]. The presence of dense αB-crystallin inclusion bodies in the central lens fiber cells of αA(-/-) mice was also observed, suggesting that αA-crystallin may be necessary for maintaining the solubility of other crystallins in the lens [12]. It was also found that the absence of αA increases cell death during the mitotic phase [13]. On the other hand, lenses in the αB(-/-) mice developed normally and were remarkably similar to wild-type mouse lenses, but αB(-/-) mice showed skeletal muscle degeneration, spine curvature and a life span one half that of wild type mice [14]. Further, lens cells from αB(-/-) mice exhibited a greater tendency for hyperproliferation in culture and genomic instability [15]. Recent studies have addressed the link between crystallin expression and progression of retinal diseases. The level of αB-crystallin in rd1 mouse retina increased significantly at 15 days postnatal, which correlated with the stage of maximal rod degeneration [16,17]. Further, an increase in αB-crystallin was also found in various retinal degenerations which varied according to the severity, type and onset of the degeneration [18]. Oxidative stress could cause alterations in crystallin content of the retina as has been observed in brain and Alzheimer disease, but the evidence is lacking at the present time [19,20].

In an attempt to better understand the role of drusen in age-related macular degeneration (AMD), Crabb et al. performed a proteomic analysis of drusen preparations from AMD and non-AMD donor eyes [21]. They found that crystallins were detected in all drusen preparations in AMD patients, and were inconsistently observed in non-AMD samples. The results indicated that αA and αB-crystallins accumulated in Bruch membrane and choroidal connective tissue to a greater degree in AMD than in normal aging [22]. It was suggested that accumulation of crystallins was a stress response manifested during the presence of AMD, and that crystallins may be involved in trapping damaged proteins and preventing their aggregation. Recent drusen analyses on primates have identified an elevated titer for μ-crystallin antibodies in macular degeneration animals, suggesting that autoantibodies against μ-crystallin may be involved in the pathogenesis of the disease [23].

The compartmental localization of α-crystallins in ocular tissues and its significance are topics of great interest. α-Crystallins are predominantly cytosolic proteins but nuclear localization of αB-crystallins has been reported [24,25]. Deretic et al. [26] had shown that αA and αB-crystallins are expressed in frog retinal cells in soluble form and associated with post-Golgi membrane of photoreceptors that participate in the transport of newly synthesized rhodopsin. Recently, studies in U373MG cells, which express appreciable amounts of αB-crystallin, showed that αB-crystallin localized in the perinuclear region of the Golgi [27]. The function of crystallins in the Golgi and the effects of physical and chemical stress on their reorganization in Golgi as well as other compartments has not been fully explored.

In the present study we have investigated the effect of the absence of α-crystallins on the susceptibility of RPE cells to apoptotic stimuli. The influence of oxidative stress on the expression and subcellular distribution of α-crystallins in human RPE is also examined.

## Methods

### Chemicals and materials

Hydrogen peroxide was obtained from Sigma Aldrich (St. Louis, MO). The 129S6/SvEvTac control mice were purchased from Taconic Farms (Germantown, NY), while the αA and αB-crystallin knockout mice in 129S6/SvEvTac background were obtained from the National Eye Institute [12,14]. Human RPE cells were isolated from fetal human donor eyes obtained from Advanced Bioscience Resources, Inc., (Alameda, CA). Eight donors were used for experiments, and donor to donor variation had a negligible effect on the results.

### Isolation and characterization of primary cultured retinal pigment epithelium from knockout mice

All animal studies were conducted with adherence to the ARVO animal statement. The cornea, lens, vitreous, and retina were removed from eyes soaked in phosphate buffered saline (PBS) containing 5% penicillin/streptomycin (Sigma). The choroid/sclera tissue was then placed into a 2% dispase solution in PBS for 20 min at 37 °C. After the incubation, the tissue was rapidly pipetted up and down for 30 s. The dispase solution containing the RPE cells was passed through a 70 μ filter followed by a 40 μ filter after which the cells were spun down and resuspended in Ham's F-12 Media (Cellgro, Herndon, VA) containing at least 25% fetal bovine serum (FBS; Irvine Scientific, Santa Ana, CA). The RPE were then grown on laminin coated plates (Becton Dickinson and Company, Franklin Lakes, NJ), and primary passages of wild type RPE controls, αA(-/-), and αB(-/-) mice were then incubated in medium containing 1% FBS overnight prior to treatment with H_2_O_2_ (100 μM -200 μM) for 24 h. RPE cell cultures were characterized by immunoreactivity for cytokeratin and RPE65, established markers of RPE.

### Cell culture and treatment for human retinal pigment epithelium

Studies using cultured human RPE were approved by the Institutional Review Board of the University of Southern California and adhere to the Declaration of Helsinki. Primary RPE cells were cultured in DMEM with 2 mM L-glutamine, 100 U/ml penicillin, 100 μg/ml streptomycin (Sigma), and 10% heat-inactivated FBS as previously described [28]. Third to fourth passage cells at >90% confluency were used in all experiments. Prior to treatment, cells were changed to 1% FBS DMEM for 16 h and then treated with varying doses of H_2_O_2_ for 24 h. Initial studies on time course of H_2_O_2_ treatment established 24 h as an optimal time point for all studies. Experiments in 1% serum were repeated in serum-free media, and subsequent results revealed similar trends. Cell viability following H_2_O_2_ treatment protocol in our experiments was verified by Trypan Blue (Cellgro), and Annexin V staining (Annexin V FLUOS staining kit; Roche Applied Science, Indianapolis, IN).

### Apoptosis assays

To determine whether knockout of α-crystallin gene expression in mouse RPE cells would cause an increase in apoptosis, DNA cleavage of αA and αB-crystallin knockout mice and wild type control cells were measured by TdT-mediated dUTP nick-end labeling (TUNEL; In Situ Cell Death Detection Kit, Fluorescein; Roche). After treatment, floating and adherent cells were collected and pooled together. The cells were then fixed with 4% paraformaldehyde in PBS (pH 7.4) followed by permeabilization with 0.1% Triton X-100 in 0.1% sodium citrate. To label DNA strand breaks, cells were incubated with 50 μl TUNEL reaction mixture containing TdT and fluorescein-dUTP in the binding buffer and incubated for 90 min at 37 °C in a humidified chamber. Cells were then washed and analyzed by flow cytometry. Further differentiation between apoptotic versus necrotic mechanisms of cell death was achieved by Annexin V and propidium iodide (PI) staining (Annexin V FLUOS staining kit; Roche) of RPE isolated from wild type and α-crystallin knockout mice at the various doses of oxidative stress. Cells were analyzed using flow cytometry. Cells positive for Annexin V staining, but not PI were gated as early apoptotic cells. AnnexinV/PI double positive cells indicated either late stage apoptosis or necrosis.

### Caspase-3 activation

Caspase-3 activation was determined using a CaspACE^TM^ FITC-VAD-FMK In Situ Marker (Promega, Madison, WI), a FITC-conjugated caspase inhibitor. Cells were collected and incubated with FITC-VAD-FMK for 60 min, then measured by flow cytometry using the FL-1 setting. Ten thousand events were recorded in each analysis.

### Mitochondrial membrane permeability transition

The loss of mitochondrial membrane potential by apoptosis is marked by increased mitochondrial permeability transition (MPT) thought to occur through the formation of pores in the mitochondria by dimerizing apoptotic proteins. During this process, the electrochemical gradient across the mitochondrial membrane collapses. To assess the mitochondrial membrane potential, a cell permeable cationic dye (Mito Flow; Cell Technology, Mountain View, CA) is added to the cells for 30 min at culture conditions and then analyzed by flow cytometry using FL-3 setting. Ten thousand events were recorded in each analysis. Healthy cells retain the reagent, while apoptotic cells exhibit a lower fluorescence signal due to less accumulation of the dye.

### Expression of α-crystallin genes with H_2_O_2_ treatment

Initial relative gene expression levels of αA and αB-crystallin was examined by semi-quantitative reverse transcriptase polymerase chain reaction (RT-PCR). cDNA from harvested RNA was transcribed using oligo(dT) and 5 μg of total RNA. 1 μl of cDNA was added to a master mix of MgCl_2_, 10X buffer, dNTP, primers (see below), and Taq polymerase (Qiagen, Valencia, CA). After 30 cycles, samples were run on an agarose gel with ethidium bromide. Quantitative expression of α-crystallin was examined using real-time PCR (LightCycler; Roche). Trizol (GIBCO BRL, Rockville, MD) was used to extract and isolate RNA, while contaminating genomic DNA was removed with a DNAase kit (DNA-free; Ambion, Austin, TX). One μg of total RNA, measured by a spectrophotometer, was added to oligo(dT)_15_ primer (Promega, Madison, WI) and AMV reverse transcriptase (Promega) for the reverse transcriptase reaction. After a 1:10 dilution of the cDNA, 2 μl of the cDNA was added to 2 μl of green fluorescent dye with Taq DNA polymerase in a 20 μl PCR mix (LightCycler FastStart DNA Master SYBR Green I, Roche). Glyceraldehyde-3-phosphate dehydrogenase (GAPDH) served as an internal control. Human primers were designed using Primer Express software (Applied Biosystems, Foster City, CA) and were only selected if the pair spanned across exons to minimize non-specific products (Applied Biosystems, Foster City, CA) and purchased from Qiagen (Valencia, CA): αA 5'-GAG ATC CAC GGA AAG CAC AAC-3' (nucleotide position 52-72) and 5'- GGT AGC GGC GGT GGA ACT-3' (107-127); αB 5'-TCC CCA GAG GAA CTC AAA GTT AAG-3' (278-301) and 5'-GGC GCT CTT CAT GTT TCC A-3' (327-347); GAPDH 5'-CCA CAT CGC TCA GAC ACC AT-3' (85-104) and 5'-GGC AAC AAT ATC CAC TTT ACC AGA GT-3' (150-169).

Product formation detection was set in the center of the linear portion of PCR amplification, and the cycle at which each reaction reached the set threshold (C_T_) was determined. Relative change in mRNA expression was calculated to obtain the ΔΔC_T_ values. Four separate sets of RNA were isolated and examined, and each set was tested in duplicate. Levels were normalized relative to GAPDH mRNA and reported as fold change over controls.

### Isolation and fractionation of proteins

For crude cell lysates, trypsinized cells were pelleted and washed once with PBS. Fifty microliters of Mammalian Protein Extraction buffer (Pierce, Rockford, IL), along with a 1:100 dilution of proteinase inhibitor mix (Sigma), was added to each pellet. After incubation for 1 h at 4 °C, cell debris was pelleted at 14,000xg for 10 min. The remaining supernatant, containing the soluble cell proteins, was measured for protein concentration, using bovine serum albumin as a standard.

To separate mitochondrial proteins from cytosolic proteins, mitochondria were first isolated using a Mitochondria/Cytosol Fractionation Kit (BioVision Inc., Mountain View, CA). To check the efficiency of homogenization, suspensions were observed under a microscope. A shiny ring around the cell indicated intact cells, and 35 strokes with a dounce homogenizer lysed 90% of the cells. The lysate was first spun for 10 min at 700xg to remove cellular debris and then at 10,000xg for 30 min to pellet the mitochondria. The resulting supernatant was saved as the cytosol portion while the pellet, containing whole mitochondria, was lysed with a mitochondria-specific buffer supplied with the kit. The purity of the fractions was checked by Western blot analysis with a polyclonal antibody against prohibitin, a mitochondrial marker (Abcam, Cambridge, MA) and GW182, a cytoplasmic resident protein (Novus Biologicals, Littleton, CO). Nuclear fractions were isolated using a Nuclear/Cytosol Fractionation Kit (BioVision Inc.). Harvested samples were examined by Western blot analysis.

### Protein analysis

Protein expression was examined using Western blot techniques. Concentration of harvested proteins was examined by the Bradford-Lowry assay. Equal amounts of protein lysate (15-50 μg) were resolved on 12.5-15% Tris-HCl polyacrylamide gels (Ready Gel; Bio-Rad, Hercules, CA) at 120 V and then transferred to a PVDF blotting membrane (Millipore, Bedford, MA). Each membrane was blocked with 5% blotting grade non-fat dry milk (Biorad), incubated with rabbit anti-αA crystallin or anti-αB crystallin antibody (Stressgen, San Diego, CA). Specificity of antibodies was confirmed by testing in knockout mice; αA showed no immunoreactive band in αA(-/-) and αB showed no immunoreactive band in αB(-/-) retina. After incubation with the secondary antibody (Vector Laboratories, Burlingame, CA), protein bands were detected by chemiluminescence (Amersham Pharmacia Biotech, Cleveland, OH). To verify equal loading of proteins, gels were stained with Coomassie stain (Bio-Safe Coomassie Stain, BioRad) or PVDF membranes were stripped with Tris-buffered saline with 0.1% Tween for 30 min at 60 °C and then incubated with GAPDH antibody (Ambion).

### Confocal microscopy

RPE cells, grown to >90% confluency on chamber slides (Lab-Tek, Naperville, IL) and exposed to oxidative stress, were examined for α-crystallin expression and for mitochondrial localization. To visualize the mitochondria, mitochondria tracker (Molecular Probes, Eugene, OR) was added to samples for 30 min, prior to fixation with 4% paraformaldehyde. GM130 antibody (BD Biosciences, San Jose, CA) was used to label the Golgi body. Cells were then permeabilized with 0.1% Triton-X100 (J. T. Baker Chemical Co, Phillipsburg, NJ) for 15 min, and then blocked with 5% blotting grade non-fat dry milk (Bio-Rad) in Tris buffered saline plus 0.1% Tween for 15 min. A 1:100 dilution of αA or αB crystallin antibody was incubated overnight at 4 °C prior to addition of Cy5-conjugated goat anti-rabbit secondary antibody (Jackson ImmunoResearch Laboratories, West Grove, PA) for 30 min the following day. Slides were examined using a Zeiss LSM510 (Zeiss,Thornwood, NY) confocal microscope.

### Transmission electron microscopy

Trypsinized cells were pelleted and fixed in 2% paraformaldehyde and 0.1% glutaraldehyde in phosphate buffer (pH 7.4) for 1 h at room temperature. The fixed pellets were dehydrated through an ethanol (EtOH) dilution series up to 100% EtOH, infiltrated in an 1:1 EtOH / LR White mixture overnight, embedded in 100% LR White acrylic resin (Ted Pella Inc., Redding CA) in beam capsules, and incubated overnight at 60 °C. The blocks were then ultra thin sectioned (75 nm in thickness) and placed on parlodian coated nickel grids. Sections on grids were etched with 0.5% sodium metaperiodate 10 min at room temperature to remove excess resin, and then washed 5 times for 10 min each by drops of 0.025 M Tris buffer (pH 7.4). Sections were placed in a blocking solution of 5% BSA in 0.025 M Tris buffer for 15 min at room temp. After addition of the primary antibody diluted with 5% BSA and 0.025 M Tris for 2 h at 37 °C, the grids were then incubated in secondary antibody conjugated to 10 nm gold (Ted Pella Inc.) in 0.025 M Tris buffer at 37 °C for 45 min and counterstained with saturated uranyl acetate and lead citrate. Analysis was performed on a Zeiss EM 10 electron microscope (Zeiss).

### Statistical analysis

Statistical analyses were performed using SAS Proc GLM procedure (SAS version 9.1, SAS Institute, Cary, NC). Different statistical strategies of multiple comparisons were used to test the differences among experimental groups. Specifically, multiple comparisons were performed on TUNEL data with Bonferroni correction and AnnexinV/PI cell death data with Tukey tests. All other data were analyzed using Dunnett's tests. Accepted level of significance for all tests was p<0.05.

## Results

### Increased susceptibility to apoptosis from H_2_O_2_ treatment in RPE from α-crystallin(-/-) mice

**Figure 1:**
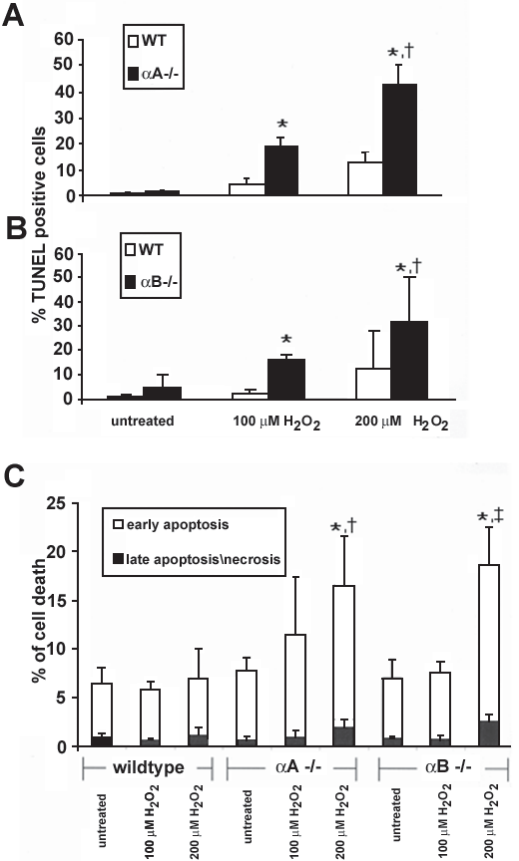
Effect of H_2_O_2_ on cell death in retinal pigment epithelium from α-crystallin (-/-) and wild type mice. RPE from knockout mice grown to confluency were treated with 100 μM or 200 μM H_2_O_2_ for 24 h and stained for apoptosis by TUNEL and quantified by flow cytometry. Cell death of RPE increased in a dose dependent manner; however, αA(-/-) (**A**) and αB(-/-) (**B**) RPE showed an increased sensitivity to apoptosis from H_2_O_2_ treatment compared to wild type cells. In the α-crystallin knockout RPE at the maximum oxidative stress used (200 μM H_2_O_2_), >85% of the dead cell population was AnnexinV+/PI- indicating an apoptotic mechanism of cell death; <15% of cells were positive for both Annexin V and PI indicating either necrosis or later stage apoptosis (**C**). Data are mean±SD (n=3-4). One and two asterisks indicate p<0.05 for the two H_2_O_2_ doses versus untreated wild type controls, and p<0.05 for dose-dependency in TUNEL positive cells in knockout mice at 100 μM and 200 μM H_2_O_2_ doses, respectively. Difference between apoptosis in untreated αA(-/-) and αB(-/-) RPE compared to wild type did not achieve statistical significance (p=0.25).

RPE cells isolated from αA(-/-), αB(-/-), and wild-type mice were treated with 100 μM or 200 μM H_2_O_2_ for 24 h. Live and dead cells were harvested and stained by TUNEL and then analyzed for TUNEL positivity by flow cytometry (Figure 1). αA(-/-) and αB(-/-)RPE showed a dose-dependent increase in apoptotic sensitivity (p<0.05, 100 μM versus 200 μM H_2_O_2_) compared to wild type cells (Figure 1A,B, respectively). Cell death with 100 μM H_2_O_2_ treatment in αA and αB knockout RPE (19% and 15%) was significantly higher than the wild type control RPE (p<0.05). Percentage of apoptotic cells showed a further increase to 42.5% and 32% in αA and αB knockout RPE with 200 μM H_2_O_2_, which was again significantly higher (p<0.05) than the corresponding controls. Cell death was also analyzed by Annexin V and PI assays. Results confirmed that cell death from H_2_O_2_ treatment in α-crystallin knockout RPE was predominantly by apoptosis (Figure 1C). In the α-crystallin knockout RPE at our maximum oxidative stress (200 μM H_2_O_2_), >85% of the dead cell population was AnnexinV^+^/PI^-^ indicating an apoptotic mechanism of cell death; <15% of cells were positive for both Annexin V and PI indicating either necrosis or later stage apoptosis. Similar to TUNEL data, αA(-/-) and αB(-/-) RPE showed a greater susceptibility to dose-dependent cell death (αA: p<0.05, αB: p<0.01), and values of knockout cells were significantly higher in comparison to wild type controls (p<0.05).

### Increased caspase-3 activation and mitochondrial membrane permeability transition in RPE from αA-crystallin(-/-) mice

**Figure 2:**
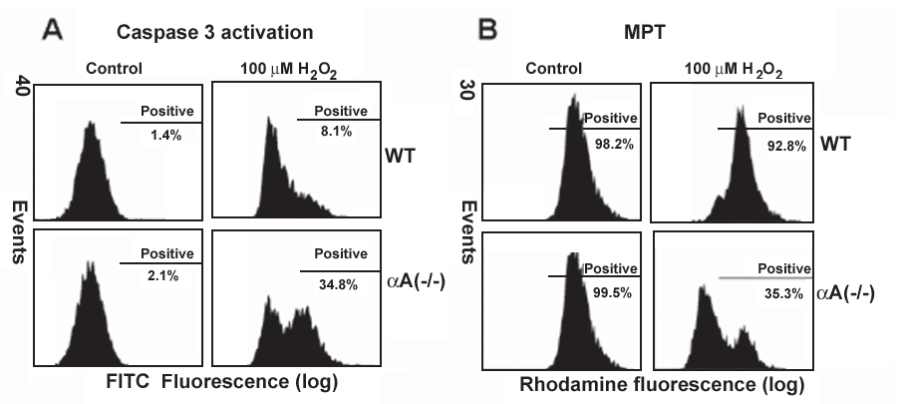
Effect of H_2_O_2_ treatment in retinal pigment epithelium from αA-crystallin(-/-) and wild type mice on caspase-3 activation and mitochondrial membrane permeability transition. **A**: Caspase-3 activation was measured by staining with FITC-VAD-FMK. αA(-/-) retinal pigment epithelium (RPE) showed an increase in caspase-3 activation compared to wild type under H2O2 treatment. **B**: αA(-/-) RPE also exhibited an increase in mitochondrial permeability transition compared to wild type RPE under H_2_O_2_ treatment.

Evidence for the increased activation of caspase-3 with H_2_O_2_ in αA-crystallin(-/-) RPE by flow cytometry is shown in Figure 2A. In flow cytometric analysis, H_2_O_2_ treatment only modestly increased the number of active caspase-3 positive cells in wild type RPE from 1.4% to 8.1% (p<0.05) while dramatically increasing the number of active caspase-3 positive cells in αA-crystallin(-/-) RPE from 2.1-34.8% (p<0.01 versus untreated and wild type controls). MPT studies also revealed an increased change in membrane potential in H_2_O_2_-treated αA(-/-) RPE compared to wild type cells (B). In wild type RPE, the MPT values by flow cytometry revealed no statistically significant changes with and without H_2_O_2_ treatment. In αA(-/-) RPE, treatment with 100 μM H_2_O_2_ resulted in a 64.2% decrease in fluorescence from untreated controls (p<0.01). Insufficient numbers of cells were available to perform parallel multiple experiments in αB(-/-) RPE; however, in one complete experiment (results not shown), we were able to demonstrate that H_2_O_2_-treated αB(-/-) RPE showed a similar response as αA(-/-) with increased caspase-3 activation and MPT. In support of our study, Kamradt et al. [7] reported that αB-crystallin suppression in human breast carcinoma cells resulted in increased caspase-3 activation.

### Changes in αB-crystallin expression in human retinal pigment epithelium with H_2_O_2_

**Figure 3:**
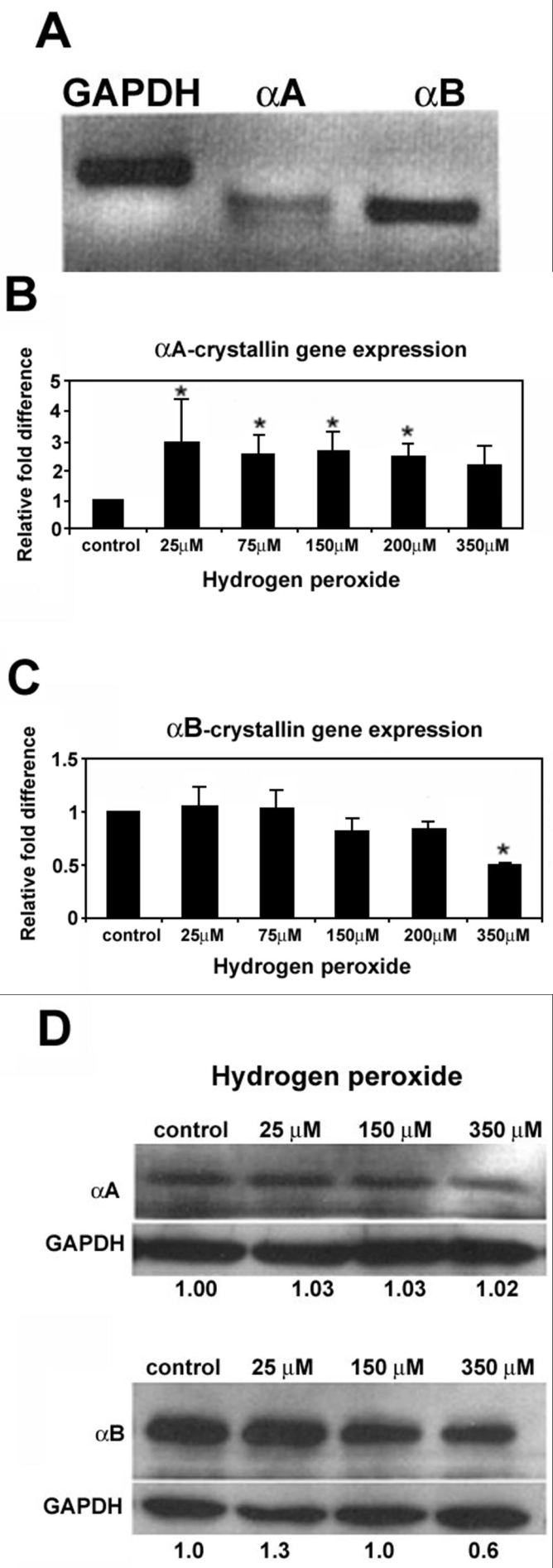
Effect of H_2_O_2_ treatment on α-crystallin protein and mRNA expression in human retinal pigment epithelium. **A**:The absolute level of αB-crystallin mRNA in RPE was much higher than that of αA-crystallin. **B**: mRNA expression after H_2_O_2_ treatment was examined by quantitative real time RT-PCR. Expression of αA-crystallin increased with treatment of H_2_O_2_; **C**: while αB-crystallin expression was biphasic with an increase with low H2O2 dose followed by a decrease at 150 μM. **D**: Protein levels were confirmed by Western blot analysis, and αB-Crystallin protein expression followed a pattern similar to its mRNA expression. Expression of αA-crystallin remained similar at different doses of oxidative stress (**D**). Data are mean±SD (n=3-4). Asterisk indicates p less than or equal to 0.05 versus untreated controls.

Initial gene expression analysis of αA-crystallin and αB-crystallin in human RPE by semi-quantitative RT-PCR showed that αB mRNA was more abundant than αA mRNA (Figure 3A). The effect of 24 h exposure to varying doses of H_2_O_2_ on αA and αB-crystallin gene expression was examined by using quantitative real time RT-PCR. αA mRNA increased with 25 μM H_2_O_2_ and remained elevated with an increase in H_2_O_2_ dose until 350 μM (Figure 3B). Gene expression of αB upon H_2_O_2_ exposure (Figure 3C) showed a dose dependent trend of decrease which reached statistically significant levels at 350 μM. It is important to note that the absolute levels of αA-crystallin in RPE are much lower than that of αB (Figure 3A, as supported by protein data in Figure 3D), and αA mRNA detection may be close to the limit of detection by multiple cycles in RT-PCR analysis.

Protein expression of αA and αB crystallin with varying doses of H_2_O_2_ was examined by Western blot analysis (Figure 3D). αA-Crystallin staining pattern showed little or no change, with and without treatment with H_2_O_2_ (Figure 3D, top). Conversely, αB-crystallin expression changed with exposure to H_2_O_2_, initially increasing at 25 μM H_2_O_2_, but decreasing in a dose-dependent manner as H_2_O_2_ dose increased to a pharmacologically toxic dose (Figure 3D, bottom).

### Changes in αB-Crystallin distribution in human retinal pigment epithelium with H_2_O_2_

**Figure 4:**
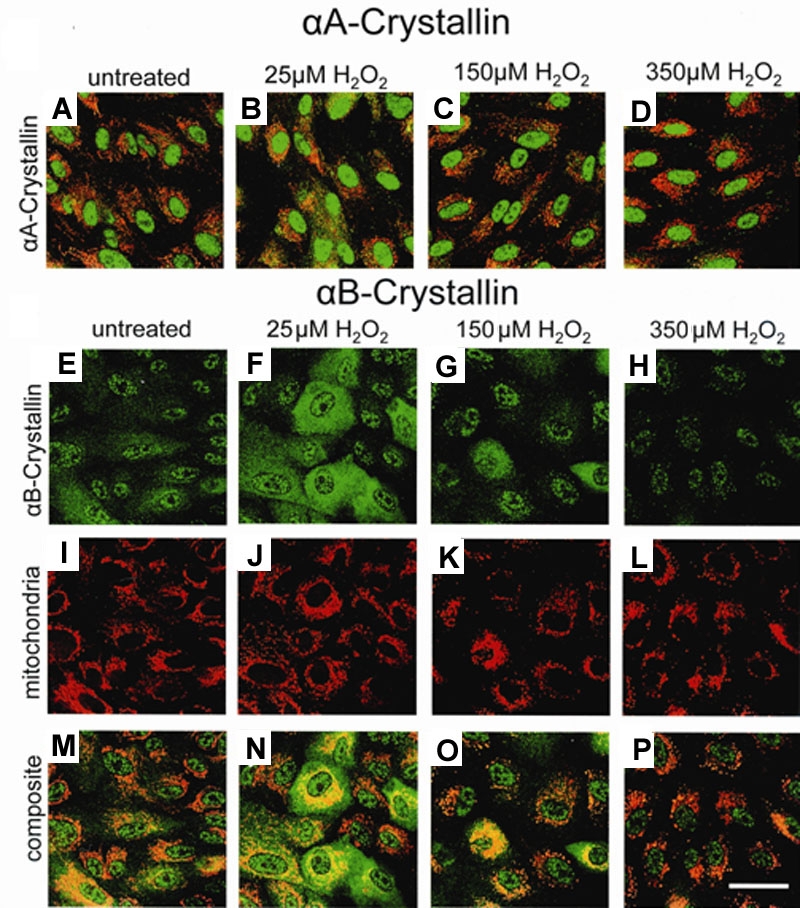
Confocal micrographs of α-crystallin cellular distribution in human retinal pigment epithelium with H_2_O_2_ treatment. Retinal pigment epithelium (RPE) grown on 4-well chamber slides were treated with 25 μM, 150 μM, or 350 μM H_2_O_2_ for 24 h and stained for either αA (**A**-**D**) or αB-crystallin (**E**-**H**). Confocal microscopy verified the Western blot analysis for both αA and αB-crystallin shown in Figure 3. Mitochondria tracker allowed visualization of mitochondria in the RPE (**A**-**D** and **I**-**L**). αA-Crystallin staining, shown as a composite image with mitochondria tracker, revealed little or no change with oxidative treatment (**A**-**D**). αB-Crystallin expression was upregulated at low doses of H_2_O_2_ as seen by the increase in fluorescent intensity (**F**). With 150 μM H_2_O_2_, αB expression decreased to control levels, but a distinct movement to perinuclear regions of the RPE is observed (**G**). αB-Crystallin fluorescence further decreased in intensity at 350 μM H_2_O_2_ (**H**). In composite images, the cellular distribution pattern of αB-crystallin was found to show a decrease in mitochondrial localization with the higher levels of oxidative stress (**M**-**P**). Bar represents 50 μm. α-Crystallin is green color, Mitochondria is red color, colocalization of α-crystallin, and mitochondria is yellow color.

**Figure 5:**
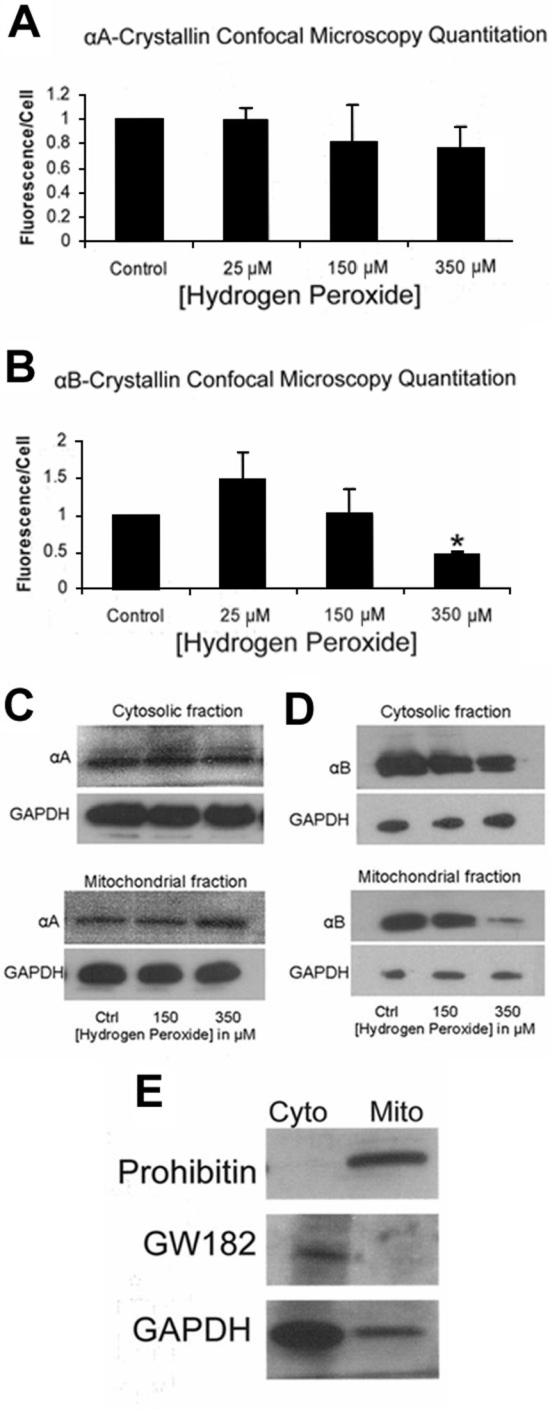
Mitochondrial distribution of α-crystallin protein in H_2_O_2_-treated retinal pigment epithelium. **A** and **B** represent quantitation of fluorescence in five experiments conducted in a similar fashion to that shown in Figure 3. Fractionated mitochondria and cytosolic proteins from H_2_O_2_-treated human retinal pigment epithelium (RPE) and controls were analyzed for α-crystallin content. Western blot analysis of αA-crystallin showed no apparent change in expression in either compartment with H2O2 exposure (**C**). αB-Crystallin in the cytosol remained relatively unchanged with varying doses of H2O2; however, mitochondrial α-crystallin decreased with increasing H2O2 dose (**D**). Purity of mitochondrial and cytosolic fractions was verified prior to crystallin analysis by two specific markers: prohibitin (**E**, top) and GM182 (**E**, middle). GAPDH, whose content is known to be much higher in cytoplasm than mitochondria, was also confirmed under our isolation conditions (**E**, bottom). Only traces (<1%) of inter-organelle contaminants were found. These fractions were then used for subsequent experiments. Asterisks indicate p<0.05 versus untreated controls. Cyto indicates cytosol; Mito indicates mitochondria.

The effect of H_2_O_2_ on αA and αB-crystallin distribution and expression in human RPE was further examined by confocal microscopy (Figure 4). RPE cells seeded on chamber slides were treated with H_2_O_2_ doses of 25 μM, 150 μM, and 350 μM. Consistent with the Western blot in Figure 3, αA-crystallin levels remained unchanged with exposure to increasing doses of H_2_O_2_, showing little or no change, with and without H_2_O_2_ treatment (Figure 4A-D). Conversely, αB-crystallin expression and distribution changed with exposure to H_2_O_2_. Twenty-five μM H_2_O_2_ exposure resulted in increased αB-crystallin protein expression in RPE, (Figure 4F), while αB-crystallin expression in RPE treated with 150 μM H_2_O_2_ (Figure 4G) declined to that of control levels (Figure 4E). The diffuse cytoplasmic pattern of αB-crystallin distribution seen in control and low H_2_O_2_ conditions shifted to perinuclear localization with the higher H_2_O_2_ dose. αB-Crystallin staining after 350 μM H_2_O_2_ treatment (Figure 4H) decreased considerably from control levels. Golgi staining (not shown) revealed no significant subcellular change in α-crystallin within the organelle. Mitochondria tracker allowed visualization of mitochondria in the RPE and effect of H_2_O_2_ exposure (Figure 4). The cellular distribution pattern of αB-crystallin showed a decrease in mitochondrial localization with increasing levels of oxidative stress (Figure 4M-P), while αA-crystallin distribution pattern remained unaltered (Figure 4A-D). Quantitation of the fluorescence per cell revealed that αB-crystallin expression increases at low doses of H_2_O_2_ (although not statistically significant) returns to control levels at 150 μM H_2_O_2_, and decreases nearly 50% from control levels at 350 μM H_2_O_2_ (p<0.05) (Figure 5B). There was no apparent change in αA-crystallin levels (Figure 5A). Isotype and "no primary antibody" controls confirmed no nonspecific fluorescence (not shown).

### Alteration of compartmental distribution of αA and αB-crystallin in retinal pigment epithelium by H_2_O_2_ treatment

Figure 4 shows the effect of varying doses of H_2_O_2_ on cytosolic and mitochondrial α-crystallin levels. Prior to analysis, mitochondrial and cytosolic fractions were checked for purity by two specific markers: prohibitin for mitochondria and GW182 for cytosol (Figure 5E top and middle, respectively). Only traces (<1%) of inter-organelle contaminants were found by densitometry of the Western blots. The purified fractions were then used for subsequent experiments. GAPDH, which is less abundant in mitochondria compared to cytoplasm [29], also served to verify fraction purity and protein quantity in mitochondria and cytosolic fractions. The results of Western blot analysis showed that H_2_O_2_ at doses of 150 mM and 350 mM H_2_O_2_ did not affect the levels of αA-crystallin significantly as compared to controls (Figure 5C), while αB-crystallin exhibited a dose-dependent decrease in mitochondrial levels in the dose range of 25-350 μM H_2_O_2_ (Figure 5D).

**Figure 6:**
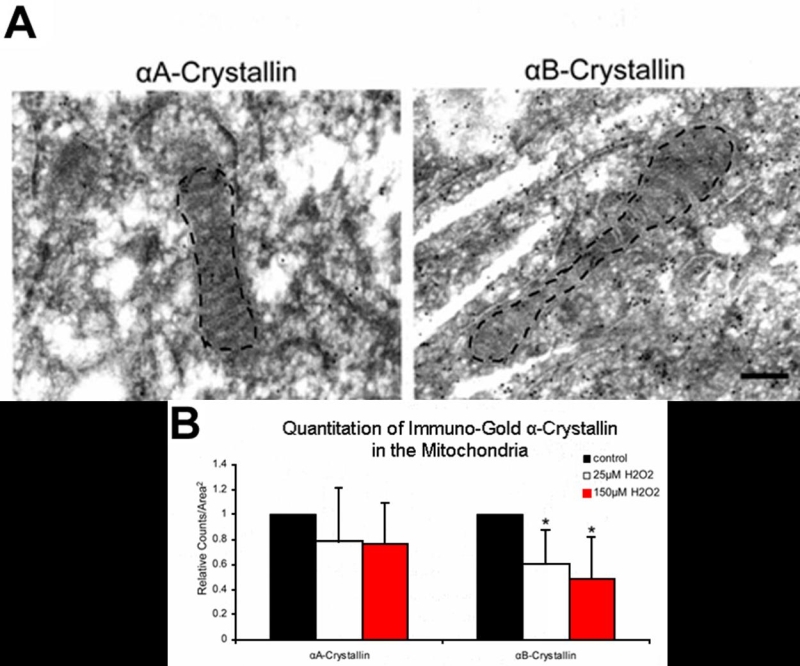
Immuno-transmission electron microscopy of gold-conjugated α-crystallin in human retinal pigment epithelium. Retinal pigment epithelium (RPE) cells treated with and without H_2_O_2_ were pelleted, fixed, sectioned and stained with 10 nm gold particles conjugated with α-crystallin antibody. Representative micrographs of untreated mitochondria in RPE labeled for αA (left) and αB-crystallin (right) are shown (**A**). Quantitation of gold particles in mitochondria revealed a dose-dependent decrease of crystallins; however, only αB-crystallin demonstrated statistical significance compared to untreated controls (**B**). Due to the variability of mitochondria size in sectioned samples, gold particle counts were normalized to untreated controls. An average of 15 micrographs was quantited for each condition. Asterisks indicate p<0.05 versus untreated controls. The scale bar represents 0.5 μm.

Immunogold-transmission electron microscopy served as an additional method to assess overall subcellular distribution and quantitation of α-crystallins in the RPE. Expression of αA and αB-crystallin was then quantitated by counting the number of 10 nm gold particles per 25 μm^2^. A slight decrease in the ratio of the number of gold particles with 25 μM (0.73) and 150 μM H_2_O_2_ (0.64) treatment to number of gold particles in the untreated controls (normalized to 1.0) was found for αA-crystallin. αB-Crystallin immunogold labeling increased by a 55% with 25 μM H_2_O_2_ (1.84) and returned to control levels with 150 μM H_2_O_2_ (1.09). The content of α-crystallins within mitochondria was then further quantitated. Figure 6A shows that immunogold labeled α-crystallin is present within outlined mitochondria in RPE cells as well as adjacent cytosol. Black dots represent gold particles localized to sites of crystallin immunoreactivity (αA: Figure 6A left panel, αB: Figure 6A right panel). Counts for gold particles demonstrated a decrease in mitochondrial αA and αB-crystallin with increasing H_2_O_2_ treatment; however, only αB values had statistical significance compared to untreated controls (Figure 6B). Due to the variability of mitochondria size in sectioned samples, gold particle counts were normalized to untreated controls. An average of 15 micrographs (approximately 300 μm^2^ of area per cell) was quantitated for each condition.

## Discussion

Our studies have demonstrated the following: (1) RPE from α-crystallin knockout mice show increased apoptotic sensitivity in response to oxidative stress from H_2_O_2_ exposure, and the extent of sensitivity to αA and αB-crystallin knockouts is similar despite marked differences in their cellular levels; (2) RPE from αA-crystallin knockout mice show increased caspase-3 activation and mitochondrial membrane permeability transition ; (3) αB-crystallin expression and distribution in human RPE changes with oxidative stress while that of αA was unchanged; (4) gene expression of αB-crystallin followed the same trend as that of protein while αA-crystallin mRNA showed an increase in expression; and (5) oxidative stress results in decreased αB-crystallin in mitochondria while αA-crystallin remains unaltered. These results reveal that both αA and αB-crystallins are critical for protection from oxidative insult but the mechanisms of action may vary between the two.

Small heat shock protein dysfunction is implicated in many diseases such as cataracts, myopathies, and a number of neuropathologies [30]. In the retina, a major function of crystallins may be to protect retinal neurons from damage by metabolic or environmental stress as seen by evidence of elevated crystallin expression in light damaged photoreceptors [31] and in models of retinal degeneration [18]. Crystallins may be important in the development of, or in response to, AMD since both αA and αB-crystallins were found to be accumulated in drusen and Bruch membrane tissues from AMD patients [22].

The magnitude and mechanisms of cellular protection from αA and αB-crystallins remain a subject of debate and has for the most part been studied in lens cells. Studies by Andley et al. (2000) [10] suggest that αA-crystallin is more potent in preventing apoptosis than αB-crystallin. In other studies, αA and αB-crystallin offered similar protection against programmed cell death; however, the two crystallins can promote cell survival by different mechanisms: αA-crystallin through the AKT pathway and αB by RAF/MEK/ERK [32]. Additional antiapoptotic mechanisms have been suggested in recent studies demonstrating interactions of α-crystallins with p53 [33,34]. It is intriguing that in our study RPE from αA(-/-) mice were just as susceptible as αB(-/-) RPE to oxidative stress, despite the relatively low abundance of αA-crystallin in RPE. Recent articles have suggested that αA-crystallin may be rendering similar degrees of protection to αB-crystallin by different signaling mechanisms [32,33].

α-Crystallins are considered to be soluble cytoplasmic proteins but have also been described in association with subcellular organelles. αB-Crystallin was shown to reside in the nucleus [25] and the perinuclear Golgi [27], and is known to associate with cellular membranes. In some instances, we found that α-crystallin expression appeared to be filamentous in appearance. A closely related protein to αB-crystallin, HSPB2, has been shown to associate with the mitochondria [35]. In this study we demonstrated the localization of α-crystallins within mitochondria and their regulation by oxidative stress. This finding is supported by a recent report by Maloyan et al. [36] showing a direct association of normal or mutant (R120G) αB-crystallin with heart mitochondria in wild type and CryAB^R120G^ mice. These authors further found that mitochondrial dysfunction is one of the earliest detectable events in the development of R120G-mediated cardiac myopathy. Furthermore, Kadono et al. [37] found that myocyte mitochondria from αB-crystallin and HSPB2 double knockout mice have increased apoptosis due to ischemia reperfusion which was accompanied by induction of MPT.

The mitochondrion is the main organelle in which oxygen metabolism occurs, and stress from H_2_O_2_ treatment increases ROS generation in human RPE [38]. We found by confocal microscopy that production of ROS was increased in both wild type [39] and αA(-/-) RPE and was localized, in part, to mitochondria (data not shown). The depletion of αB-crystallin in mitochondria with supra-physiological H_2_O_2_ doses (>300 μM) in human RPE may reflect the consumption of αB-crystallin for scavenging large amounts of ROS generated.

We found that αA-crystallin protein expression remained stable, while αB-crystallin expression increased upon low doses of H_2_O_2_ and returned back to control levels with high doses. αA-Crystallin mRNA expression showed approximately a two-fold induction with both low and high doses of oxidative stress. αB-Crystallin mRNA expression was also upregulated with low doses of H_2_O_2_, but interestingly, decreased dramatically at higher doses. The concentrations of H_2_O_2_ in this study are in the range previously used by others to examine αB-crystallin gene expression. Alge et al. [9] reported that 200 μM H_2_O_2_ resulted in an upregulation of αB-crystallin mRNA. The maximal increase occurred at 6 h in their study, while the conditions we have chosen are intended to mimic chronic oxidative stress more frequently seen in vivo. The upregulation of several genes with sublethal doses of H_2_O_2_ and a down-regulation with higher doses has been reported in a number of recent studies supporting the idea that H_2_O_2_ may act as a signaling molecule at low doses [40,41]. The exact signaling mechanism associated with H_2_O_2_ remains to be elucidated. It is worth noting that it was found that human *bcl2* gene attenuates H_2_O_2_-induced apoptosis in rabbit lens epithelial cells through down-regulation of the αB-crystallin gene [42].

Studies on crystallins have focused predominantly in the lens, where both αA and αB-crystallin are expressed in equal amounts and comprise a huge portion of total soluble protein [2]. On the other hand, in mouse retina, αB-crystallin is 15 to 30 fold lower than αA-crystallin, but distributed in the same layers [8]. While the information on α-crystallin expression in the RPE is scarce, data from our current study indicate a much higher expression of αB-crystallin as compared to αA-crystallin. RPE isolated from αA or αB-crystallin knockout mice showed an increased susceptibility to apoptosis via oxidative stress. The extent of cell death from H_2_O_2_ was similar in both αA(-/-) and αB(-/-) RPE, but it is interesting that the utilization of antiapoptotic mechanistic pathways could vary among the three prominent members of the sHSP family, namely Hsp27, αA and αB-crystallins [11,43-45].

The BCL-2 family contributes to the regulation of the swelling of the mitochondria and the opening of the permeability transition pore. Our studies have revealed that RPE cells from αA(-/-) mice show an increased mitochondrial membrane permeability transition compared to wildtype cells under H_2_O_2_ insult. While BCL-2 family members are thought to be the sentinels of cell death [46], crystallins may be acting as chaperones or regulators, possibly by interaction at the BH3 domain of these proteins. α-Crystallins may also be directly and/or indirectly affecting downstream death effectors at the caspase level. H_2_O_2_-treated RPE cells from αA(-/-) show increased caspase-3 activation compared to wildtype RPE. Studies done by others show that αB-crystallin negatively regulates cyt c and caspase-8-independent activation of caspase 3 by inhibiting its autoproteolytic maturation, which may provide further clues [7]. Further work will be needed to assess the contribution of mitochondrial (and extramitochondrial) pathways of apoptosis in RPE cells under oxidative stress.
